# Underweight and associated factors among children under age of five in low and lower-middle income African countries: hierarchical analysis of demographic and health survey data

**DOI:** 10.3389/fpubh.2024.1423603

**Published:** 2024-09-09

**Authors:** Tadesse Tarik Tamir, Alebachew Ferede Zegeye, Belayneh Shetie Workneh, Enyew Getaneh Mekonen

**Affiliations:** ^1^Department of Pediatrics and Child Health Nursing, School of Nursing, College of Medicine and Health Sciences, University of Gondar, Gondar, Ethiopia; ^2^Department of Medical Nursing, School of Nursing, College of Medicine and Health Sciences, University of Gondar, Gondar, Ethiopia; ^3^Department of Emergency and Critical Care Nursing, School of Nursing, College of Medicine and Health Sciences, University of Gondar, Gondar, Ethiopia; ^4^Department of Surgical Nursing, School of Nursing, College of Medicine and Health Sciences, University of Gondar, Gondar, Ethiopia

**Keywords:** underweight, factors, children, low, lower middle, income, Africa

## Abstract

**Introduction:**

Globally, nearly half of all deaths among children under the age of five are linked to undernutrition. These tragic outcomes are most prevalent in low- and middle-income countries. The far-reaching impact of malnutrition affects not only individuals but also their families, communities, and entire nations. By examining underweight, we gain valuable insights into the intricate network of factors influencing child health. Therefore, this study aims to assess underweight prevalence and its associated factors among under-five children in low and lower-middle-income African countries.

**Method:**

We conducted a secondary analysis of standard demographic and health surveys in 30 low and lower-middle-income African countries spanning from 2012 to 2022. Our analysis included a total sample of 200,655 children under the age of 5 years. We employed a three-level hierarchical model to assess the determinants of underweight among children in this age group. Measures of association were evaluated using adjusted odds ratios with a 95% confidence interval. Explanatory variables with a *p*-value less than the level of significance (0.05) were considered statistically significant.

**Result:**

The pooled prevalence of underweight among children under the age of five in low and lower-middle income African countries was estimated at 17.60%, with a 95% confidence interval (CI) ranging from 17.44 to 17.77%. The hierarchical analysis identified several factors significantly associated with underweight, including male gender, birth size, maternal body mass index, maternal educational level, household wealth index, antenatal care (ANC) visits, community poverty level, and income level of countries.

**Conclusion:**

The high prevalence of underweight among children under the age of five in low and lower-middle income African countries underscores the need for targeted interventions. By addressing individual, community, and country-level factors, we can work toward improving child nutrition and well-being.

## Introduction

Underweight refers to a low weight for age (weight-for-age less than −2 standard deviations (SD) of the WHO Child Growth Standards median) that can be a result of stunting, wasting, or frequently both (1–3). Globally, about half of the deaths among under-five children are related to undernutrition (4, 5). These primarily happen in low- and middle-income countries (4, 5). In terms of development, economics, social issues, and medicine, the worldwide burden of malnutrition has serious and enduring repercussions on individuals as well as their families, communities, and nations (1, 3, 5). Poor eating habits and inadequate nutrition are two of the biggest risk factors for chronic illnesses worldwide. Diabetes, several cancers, and cardiovascular disorders (heart attacks, strokes, and high blood pressure) are prominent instances of non-communicable diseases (NCDs) linked to malnutrition (5).

Malnutrition is one of the main agenda items for Sustainable Development by 2030—in particular, Sustainable Development Goal (SDG) 2 (set a concrete aim to end hunger, achieve food security and improved nutrition, and promote sustainable agriculture) and SDG 3 (to ensure healthy lives and promote wellbeing for all at all ages) (5, 6). However, undernutrition rates have increased globally since 2014 (7). In The State of Food Security and Nutrition in the World 2023, the Food and Agriculture Organization (FAO) of the United Nations reported that there were 122 million more undernourished people than there were in 2019—nearly 735 million people, or 9.2% of the world’s population (7). Most live in countries where food insecurity is pervasive. Undernutrition increased in frequency in Africa from 19.4% in 2021 to 19.7% in 2022 (7). About 45 million children under the age of five were wasted and 149 million stunted globally in 2022 (4, 5). Underweight is a direct composite indicator both chronic (stunting) and acute (wasting) undernutrition (8). Around 17.1% of children under five in the WHO’s Africa area were underweight in 2018, which corresponds to 29 million children (9, 10). These numbers represent real children facing nutritional challenges, and addressing their needs is essential for their health and well-being.

According to the evidence (11–13), the following are among the factors significantly associated with underweight among children under five: age, gender, place of residence, maternal educational levels, diarrhea, maternal occupational status, twin or triplet birth, birth order, birth weight, place of delivery, and the use of fortified baby food. In addition, this study aimed to determine individual, community, and country-level factors associated with underweight among under-five children.

Childhood underweight is a critical issue, especially in low and lower-middle-income countries (14). By studying this phenomenon, we gain insights into the complex web of factors affecting child health. Addressing childhood underweight is essential for building human capital, aligning with the Sustainable Development Goals (SDGs), and tailoring evidence-based interventions (15). Overall, studying childhood underweight in Africa lays the groundwork for targeted interventions, poverty reduction, and healthier futures for children. Hence, this study aimed to assess the underweight and associated factors among under-five children in low and lower-middle-income African countries using data from standard demographic and health surveillance data.

## Methods

### Data source, setting and sampling

This study conducted a secondary analysis of standard demographic and health surveys in low and lower-middle-income African countries from 2012 to 2022. The research utilized datasets known as the children’s recode (KR), which were obtained from the Monitoring and Evaluation to Assess and Use Results Demographic and Health Survey (MEASURE DHS) program. These datasets are publicly accessible at https://www.dhsprogram.com. The MEASURE DHS program offers researchers valuable data for formal requests related to project registration and submission. For this study, we selected a total of 30 low- and lower-middle-income African countries. The criteria for inclusion were the availability of Demographic and Health Surveys (DHS) data spanning the period from 2012 to 2022, as well as the availability of WHO weight-for-age Growth Standard scores within their DHS datasets.

The DHS data collection process employs a stratified two-stage cluster sampling technique. In the first stage, we select enumeration areas (also known as sampling clusters) based on their population size, using probability proportional to size. In the second stage, we choose a sample of households within these clusters, collecting actual data. As a result, the DHS data exhibit a hierarchical structure. Our analysis included a total sample of 200,655 children under the age of 5 years.

### Variables and data management

The outcome variable of this study was underweight among children under age of five which was determined as weight-for-age less than −2 standard deviations of the WHO Child Growth Standards median (1). It was dichotomized as “yes” for children who were underweight and “no” for children who were not underweight.

**Figure 1:**
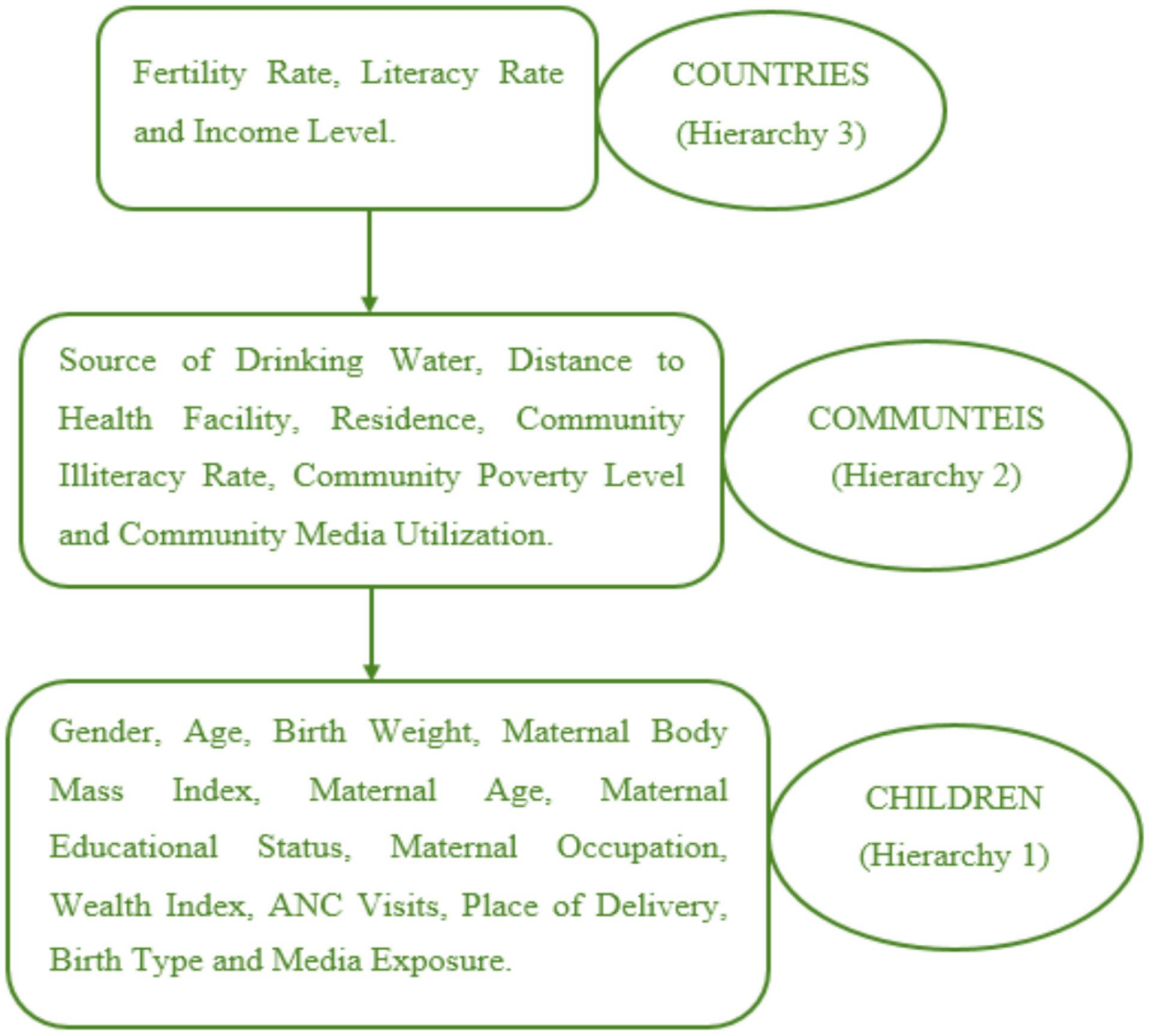
Hierarchy of variables of the study.

The study examined three hierarchical levels of independent variables. The first hierarchy included gender, age, birth weight, maternal body mass index, maternal age, maternal educational status, maternal occupation, wealth index, antenatal care (ANC) visits, place of delivery, birth type, and media exposure. The second hierarchy encompassed variables such as the source of drinking water, distance to health facilities, residence, community illiteracy rate, community poverty level, and community media utilization. Lastly, the third hierarchy consisted of fertility rate, literacy rate, and income level (as shown in Figure 1). Additionally, this study controlled for the effect of different survey years by including the year of the Demographic and Health Survey (DHS) as a control variable.

The World Bank classifies all countries’ economies into four income groups: low, lower-middle, upper-middle, and high-income. The thresholds for these classifications are updated annually based on the Gross National Income (GNI) *per capita* calculated using the World Bank Atlas method (16). For the 2022 fiscal year, which represents the most recent classification, low-income countries are defined as those with a GNI *per capita* of $1,165 or less (17). In contrast, lower-middle-income countries are defined as those with a GNI *per capita* between $1,166 and $4,565 (16, 17). This income-based categorization provides a standardized framework for analyzing economic development and comparing the relative prosperity of different nations around the world (16, 17).

The children’s recode data archive, available in Stata format for all countries, was extracted from the MEASURE DHS website, https://www.dhsprogram.com. After extraction, individual country datasets were combined, resulting in a single data file created using Stata version 14. To ensure valid conclusions, weighting was applied, accounting for the sampling design. The study’s findings were then presented in tables and charts, reflecting weighted frequencies and percentages.

### Statistical analysis

The analysis in this study incorporated three levels of explanatory variables using a hierarchical model. We sequentially fitted five models: First, the null model: This model included only the outcome variable without any explanatory variables. Second, Model I: We incorporated the outcome variable, first-level explanatory variables, and a control variable. Thirdly, Model II: This model included the outcome variable, second-level explanatory variables, and the control variable. Then, Model III: We extended the model to include the outcome variable, third-level explanatory variables, and the control variable. Finally, in Model IV, we integrated all levels of explanatory variables, the control variable, and the outcome. In our assessment, we considered both random and fixed effects within these models.

In terms of the random effect structure, given the cross-sectional nature of the DHS data, we exclusively employed a random intercept model. Within this model, we calculated the variance, intra-class correlation coefficient, and median odds ratio. Moving to the fixed component of the model, we evaluated the adjusted odds ratio at a 95% confidence interval. Explanatory variables with a *p*-value less than the level of significance (0.05) were considered statistically significant. Deviance was used for model comparison, due to nested nature of the hierarchical mode.

The three-level hierarchical (multilevel) model, designed to account for the hierarchical data with three levels, is represented as (18, 19);

yijk=β0k+β1kxijk+rjk+uik+eijk

Where yijk is the dependent variable for the i^th^ individual in the j^th^ community in the k^th^ country, β0k and β1k are the intercept and slope coefficients for the k^th^ country, xijk is the independent variable for the i^th^ individual in the j^th^ community in the k^th^ country, rjk is the random effect for the j^th^ community in the k^th^ country, uik is the random effect for the i^th^ individual in the k^th^ country and eijk is the residual error for the i^th^ individual in the j^th^ community in the k^th^ country.

## Results

The study included a total of 200,655 children under the age of 5, with 50.43% males and 49.57% females. More than half (57.16%) of the participants were 24 months of age or older. Out of these children, 99,865 (61.40%) were born to mothers with a normal body mass index. The majority (70.63%) of the study subjects were born to mothers aged 20–34 years, while approximately 41% had mothers with no formal education. Additionally, 57.41% of the subjects’ mothers had four or more antenatal care (ANC) visits during pregnancy. A bit less than two-thirds (62.07%) of the study participants were from communities where distance to health facilities was not a problem. Furthermore, 138,875 (69.21%) subjects resided in rural areas, and over half (52.26%) came from low-income countries.

Regarding underweight status, 18,879 (18.66%) of male and 16,440 (16.53%) of female participants were underweight. Additionally, 7,762 (12.56%) of urban and 27,557 (19.84%) of rural resident children were underweight. Furthermore, 22,225 (21.20%) children in low-income countries and 13,094 (13.67%) children in lower-middle-income countries exhibited underweight conditions (Table 1).

**Table 1:** Description of underweight by hierarchy of variables in low and lower-middle income African countries (N = 200,655).

Variables at respective hierarchy		Frequency (Percent)	Underweight	
		*N* (%)	Yes [*N* (%)]	No [*N* (%)]
Individual level variables				
Gender	Male	101,181 (50.43)	18,879 (18.66)	82,302 (81.34)
	Female	99,474 (49.57)	16,440 (16.53)	83,034 (83.47)
Age	0–23 months	63,530 (42.84)	9,592 (15.10)	53,938 (84.90)
	24–59 months	84,758 (57.16)	14,030 (16.55)	70,728 (83.45)
Birth size	Small	95,382 (52.59)	22,600 (23.69)	72,782 (76.31)
	Average	73,663 (40.61)	9,047 (12.28)	64,616 (87.72)
	Large	12,339 (6.80)	1,179 (9.56)	11,160 (90.44)
Maternal BMI	Low	16,409 (10.09)	5,734 (34.94)	10,675 (65.06)
	Normal	99,865 (61.40)	19,626 (19.65)	80,239 (80.35)
	High	46,362 (28.51)	4,243 (9.15)	42,119 (90.85)
Maternal age	15–19	11,335 (5.65)	2,101 (18.54)	9,234 (81.46)
	20–34	141,728 (70.63)	24,551 (17.32)	117,177 (82.68)
	35–49	47,592 (23.72)	8,667 (18.21)	38,925 (81.79)
Maternal education	No formal education	82,515 (41.12)	20,201 (24.48)	62,314 (75.52)
	Primary	59,253 (29.53)	9,367 (15.81)	49,886 (84.19)
	Secondary	49,681 (24.76)	5,182 (10.43)	44,499 (89.57)
	Higher	9,206 (4.59)	569 (6.18)	8,637 (93.82)
Wealth index	Poorest	49,383 (24.61)	11,060 (22.40)	38,323 (77.60)
	Poorer	41,944 (20.90)	8,103 (19.32)	33,841 (80.68)
	Middle	40,481 (20.17)	7,010 (17.32)	33,471 (82.68)
	Richer	37,061 (18.47)	5,700 (15.38)	31,361 (84.62)
	Richest	31,786 (15.84)	3,446 (10.84)	28,340 (89.16)
ANC visits	No visits	17,343 (12.57)	5,010 (28.89)	12,333 (71.11)
	1–3 visits	41,429 (30.03)	7,554 (18.23)	33,875 (81.77)
	4 or more	79,209 (57.41)	10,331 (13.04)	68,878 (86.96)
Place of delivery	Home	65,166 (34.93)	17,068 (26.19)	48,098 (73.81)
	Health facility	121,380 (65.07)	16,356 (13.48)	105,024 (86.52)
Birth type	single	194,382	33,525 (17.25)	160,857 (82.75)
	multiple	6,273	1,794 (71.40)	4,479 (28.60)
Media exposure	Yes	122,624 (61.24)	16,531 (13.48)	106,093 (86.52)
	No	77,600 (38.76)	18,665 (24.05)	58,935 (75.95)
Community level variables				
Source of water	Improved	130,692 (65.13)	20,872 (15.97)	109,820 (84.03)
	Unimproved	69,963 (34.87)	14,447 (20.65)	55,516 (79.35)
Distance to health facility	Not big problem	107,347 (62.07)	15,736 (14.66)	91,611 (85.34)
	Big problem	65,596 (37.93)	12,213 (18.62)	53,383 (81.38)
Residence	Urban	61,780 (30.79)	7,762 (12.56)	54,018 (87.44)
	Rural	138,875 (69.21)	27,557 (19.84)	111,318 (80.16)
Community women illiteracy	Low	84,841 (42.28)	12,387 (14.60)	72,454 (85.40)
	High	115,813 (57.72)	22,932 (19.80)	92,881 (80.20)
Community media utilization	Low	125,553 (62.57)	24,537 (19.54)	101,016 (80.46)
	High	75,102 (37.43)	10,782 (14.36)	64,320 (85.64)
Community poverty	Low	91,177 (45.44)	15,209z (16.68)	75,968 (83.32)
	High	109,471 (54.56)	20,108 (18.37)	89,363 (81.63)
Country level variables				
Fertility rate	Low	132,806 (66.19)	20,884 (15.73)	111,922 (84.27)
	high	67,849 (33.81)	14,435 (21.28)	53,414 (78.72)
Literacy rate	Low	106,340 (53.00)	14,631 (13.76)	91,709 (86.24)
	High	94,315 (47.00)	20,688 (21.94)	73,627 (78.06)
Income	Low	104,859 (52.26)	22,225 (21.20)	82,634 (78.80)
	Lower middle	95,796 (47.74)	13,094 (13.67)	82,702 (86.33)

ANC, Antenatal Care; BMI, Body Mass Index.

### Prevalence of underweight among under five children in low and lower-middle income African countries

**Figure 2:**
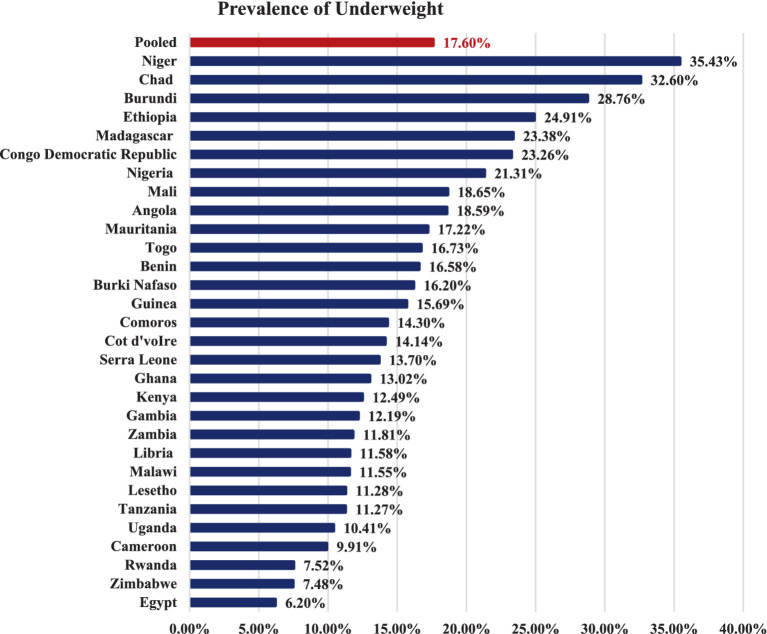
Prevalence of underweight among under five children in low and lower middle income African countries.

The pooled prevalence of underweight among children under the age of five in low and lower-middle income African countries was determined to be 17.60%, with a 95% confidence interval (CI) ranging from 17.44 to 17.77%. Notably, Niger exhibited the highest prevalence of underweight (35.43%), while Egypt had the lowest prevalence (6.20%) (Figure 2).

### Random effect and model comparison

In the null model of the random effect model, we observed significant variation in underweight among children within communities (τ = 0.280, *p* < 0.001) and across countries (τ = 0.457, *p* < 0.00). This finding suggests that a hierarchical (multilevel) model is appropriate for analyzing the data. Specifically, the intra-community and intra-country correlation coefficients in the null model indicate that 7.84 and 12.25% of the total variation in underweight among children can be attributed to variations between communities and countries, respectively.

The unexplained heterogeneity (MOR) in underweight among communities and countries, without considering explanatory variables, was 1.65 and 1.90, respectively. However, after incorporating a full hierarchy of factors, the heterogeneity (MOR) decreased to 1.31 and 1.26. Model IV, which exhibited the smallest deviance value, was selected as the best-fitting model (Table 2).

**Table 2:** Random effect and model comparison of underweight among under five children in low and lower-middle income African countries.

Parameters		Null model	Model I	Model II	Model III	Model IV
Random effect						
Variance (τ)	Country	0.457**	0.094*	0.186**	0.173**	0.081*
	Community	0.280**	0.073*	0.114**	0.106**	0.061*
ICC (%)	Country	12.25	2.78	5.35	5.00	2.40
	Community	7.84	2.17	3.35	3.12	1.82
MOR	Country	1.90	1.34	1.51	1.48	1.31
	Community	1.65	1.29	1.37	1.36	1.26
Model comparison						
Log likelihood		−92776.47	−30421.43	−75106.54	−90626.64	−30398.18
Deviance		185,552.94	60,842.86	150,213.08	181,253.28	60,796.36

ICC, Intra-community and intra-country correlation coefficients; MOR, Median Odds Ratio.

### Fixed effect of underweight among under five children in low and lower-middle income African countries

The hierarchical analysis of this study found that gender, birth size, maternal BMI, maternal educational level, household wealth index, ANC visit, community poverty level, and income level of countries were significantly associated with underweight.

In this respect, the odds of being underweight were 1.29 times higher among male children (AOR = 1.29 at 95% CI: 1.24, 1.34) as compared to females. The odds of childhood underweight were 1.61 times higher among children with smaller sizes (AOR = 1.61 at 95% CI: 1.53, 1.70) than children with average sizes at birth. In addition, the odds of being underweight decreased by 39% for children of large birth size (AOR = 0.61 at 95% CI: 0.54, 0.67) compared to children with average weight. Likewise, the odds of underweight were 1.94 times higher among children born to mothers with a low body mass index (AOR = 1.94 at 95% CI: 1.83, 2.06) and reduced by 30% for children born to mothers with a high body mass index (AOR = 0.60 at 95% CI: 0.57, 0.64).

When compared to children born to mothers with a higher educational level, the odds of being underweight were 1.85, 1.44, and 1.27 times higher among children born to mothers with no formal education (AOR = 1.85 at 95% CI: 1.59, 2.16), primary schooling (AOR = 1.44 at 95% CI: 1.23, 1.67), and secondary schooling (AOR = 1.27 at 95% CI: 1.09, 1.47), respectively.

Regarding the wealth index, the odds of underweight were 1.10, 1.24, 1.34, and 1.44 times higher among children of households with richer (AOR = 1.10 at 95% CI: 1.02, 1.20), middle (AOR = 1.24 at 95% CI: 1.13, 1.35), poor (AOR = 1.34 at 95% CI: 1.22, 1.47) and poorer (AOR = 1.44 at 95% CI: 1.31, 1.58), respectively. The odds of underweight were 1.16 and 1.08 times higher among children who were born to mothers who had no ANC visits (AOR = 1.16 at 95% CI: 1.08, 1.24) and 1–3 ANC visits (AOR = 1.08 at 95% CI: 1.03, 1.13), respectively.

In addition, the odds of being underweight were 1.09 times higher among children born in a community with a high poverty level (AOR = 1.09, 95% CI: 1.03, 1.13). Moreover, the odds of being underweight were 1.11 times higher among children in countries with low income (AOR = 1.11 at 95% CI: 1.06, 1.16) (Table 3).

**Table 3:** Fixed effect of underweight among under five children in low and lower-middle income African countries.

Factors at respective hierarchy		Model I AOR (95% CI)	Model II AOR (95% CI)	Model III AOR (95% CI)	Model IV AOR (95% CI)
Survey year	2012–2014	1.28 (1.10, 1.31)	1.37 (1.31, 1.43)	1.57 (1.52, 1.62)	1.24 (1.15, 1.30)
	2015–2019	1.15 (1.10, 1.20)	1.20 (1.17, 1.24)	1.14 (1.11, 1.18)	1.13 (1.08, 1.18)
	2020–2022	1	1	1	1
Gender	Male	1.29 (1.24, 1.35)			1.29 (1.24, 1.34)*
	Female	1			1
Age	0–23 months	1.05 (0.89, 1.10)			1.03 (0.87, 1.07)
	24–59 months	1			1.0
Birth size	Small	1.64 (1.56, 1.73)			1.61 (1.53, 1.70)*
	Average	1			1
	Large	0.60 (0.54, 0.67)			0.61 (0.54, 0.67)*
Maternal BMI	Low	1.94 (1.83, 2.06)			1.94(1 0.83, 2.06)*
	Normal	1			1
	High	0.61 (0.57, 0.64)			0.60 (0.57, 0.64)*
Maternal age	15–19	0.98 (0.90, 1.06)			0.99 (0.91, 1.07)
	20–34	1			1
	35–49	1.05 (1.00, 1.10)			1.03 (0.97, 1.09)
Maternal education	No formal education	1.93 (1.66, 2.25)			1.85 (1.59, 2.16)*
	Primary	1.44 (1.24, 1.67)			1.44 (1.23, 1.67)*
	Secondary	1.26 (1.09, 1.47)			1.27 (1.09, 1.47)*
	Higher	1			1
Wealth index	Poorest	1.37 (1.26, 1.49)			1.44 (1.31, 1.58)*
	Poorer	1.30 (1.19, 1.41)			1.34 (1.22, 1.47)*
	Middle	1.21 (1.11, 1.31)			1.24 (1.13, 1.35)*
	Richer	1.09 (1.01, 1.18)			1.10 (1.02,1.20)*
	Richest	1			1
ANC visits	No visits	1.16 (1.08, 1.25)			1.16 (1.08, 1.24)*
	1–3 visits	1.07 (1.03, 1.13)			1.08 (1.03, 1.13)*
	4 or more	1			1
Place of delivery	Home	0.99 (0.94, 1.05)			1.00 (0.94, 1.06)
	Health facility	1			1
Birth type	Single	1			1
	Multiple	1.10 (1.05, 1.15)			1.05 (0.95, 1.11)
Media exposure	Yes	1			1
	No	1.06 (1.02, 1.11)			0.97 (0.73, 1.12)
Number of households	2–3	0.92 (0.86, 0.99)			0.94 (0.88, 1.01)
	4–5	0.95 (0.90, 0.99)			0.96 (0.91, 1.01)
	6 or more	1			1
Source of water	Improved		1.21 (1.18, 1.25)		1.03 (0.98, 1.07)
	Unimproved		1		1
Distance to health facility	Not big problem		1		1
	Big problem		1.09 (1.05, 1.14)		1.03 (0.94, 1.06)
Residence	Urban		1		1
	Rural		1.54 (1.49, 1.59)		1.01 (0.95, 1.07)
Community women illiteracy	Low		1		1
	High		1.34 (1.27, 1.42)		1.03 (0.97, 1.08)
Community media utilization	Low		1.49 (1.40, 1.59)		0.99 (0.94, 1.05)
	High		1		1
Community poverty level	Low		1		1
	High		1.12 (1.06, 1.18)		1.09 (1.03, 1.13)*
Fertility rate	Low			1	1
	high			1.28 (1.25, 1.32)	1.04 (0.99, 1.11)
Literacy rate	Low			1.58 (1.54, 1.62)	1.04 (0.99, 1.08)
	High			1	1
Income	Low			1.37 (1.33, 1.41)	1.11 (1.06, 1.16)*
	Lower middle			1	1

ANC, Antenatal Care; AOR, Adjusted Odds Ration; BMI, Body mass index; CI, Confidence Interval; *Statistically significant (*p*-value < 0.05).

## Discussion

Addressing childhood underweight is essential for building human capital, aligning with the Sustainable Development Goals (SDGs), and tailoring evidence-based interventions (15). Studying childhood underweight in Africa lays the groundwork for targeted interventions, poverty reduction, and healthier futures for children. Hence, this study assessed the underweight and associated factors among under-five children in low and lower-middle-income African countries using data from standard demographic and health survey.

The pooled prevalence of underweight among children under the age of five in low and lower-middle income African countries was determined to be 17.60%, with a 95% confidence interval (CI) ranging from 17.44 to 17.77%. The prevalence of underweight among children under the age of five in this study was slightly higher than the prevalence of underweight children under the age of five in the WHO’s Africa region (17.1%) (9, 10). Additionally, the prevalence in our study was far higher than the global prevalence of childhood underweight, which was 13.4% (9, 10, 20). The observed discrepancy may be attributed to the inclusion of low and lower-middle-income African countries in this study, whereas subsequent studies estimated underweight prevalence without considering the income levels of the countries. This finding highlights the higher prevalence of underweight children in economically disadvantaged African nations, emphasizing the need for global strategies to prioritize a country’s income status when implementing childhood nutritional interventions.

This study revealed that gender, birth size, maternal BMI, maternal educational level, household wealth index, ANC visit, community poverty level, and income level of countries were significantly associated with underweight.

In this regard, the odds of underweight were high among male children than females. This finding is in agreement with previous evidences (21–23). On the other hand, a study in Pakistan reported that the likelihood of a girl child being underweight is greater than that of a boy child being underweight (12). The higher likelihood of underweight among male children compared to females can be attributed to several factors. First, biological differences play a significant role. Boys tend to have a slightly higher metabolic rate than girls, which means they may burn calories faster. If their nutritional intake is insufficient, this can lead to an underweight status (24). Additionally, boys often experience growth spurts during adolescence, requiring additional energy and nutrients. If their diet does not meet these demands, they may become underweight (24). Secondly, health conditions and infections also play a role. Boys are often more susceptible to infections, such as respiratory or gastrointestinal infections, which can affect their appetite and nutrient absorption. Additionally, certain chronic illnesses (like chronic diarrhea or malabsorption disorders) can impact boys’ weight gain (24). However, we could not ignore the cultural and social factors that contribute to disparity enabling males to be nourished than females. Gender norms, shaped by societal expectations, influence feeding practices (25, 26). For instance, boys might be encouraged to eat more or prioritize certain foods, while girls may receive smaller portions. Moreover, food distribution within households can impact boys’ overall nutrition. In some families, male members receive preferential treatment when it comes to food allocation (25).

Regarding the birth size of children, the odds of childhood underweight were higher among children with smaller sizes than children with average sizes at birth. In addition, children of large birth size had lower odds of being underweight than children of average weight. This finding is in line with existing evidences (27–31). There are multiple ways in which birth size and childhood underweight are interconnected. Contributing factors include changes in epigenetic patterns, inadequate maternal nutrition during pregnancy, intrauterine growth restriction (IUGR), and placental insufficiency (32). A child’s growth is influenced by nutrition, breastfeeding practices, and overall postnatal care. Babies born with low birth weights may struggle to reach their ideal growth (33). The nutritional status of the infant was significantly influenced by the child’s size at delivery, since low birth size is indicative of controlled intrauterine development (30, 34).

Likewise, the likelihood of childhood underweight was higher among children born to mothers with a low body mass index and lower for children born to mothers with a high body mass index. Studies have also witnessed the same (31, 35, 36). Proper nutrition is crucial for women during both prenatal and postnatal phases to support healthy child growth. Maternal body mass index (BMI) plays a significant role in determining child undernutrition, and it is influenced by maternal nutrition. Children born to healthy mothers are at a reduced risk of being undernourished (35).

Coherent with previous findings (12, 37, 38), this study found that no or lower level of maternal education was associated with higher odds of childhood underweight. The association could be due to the fact that educated mothers tend to have better knowledge about nutrition, hygiene, and child care practices (39). They also have improved access to resources such as nutritious food and healthcare. Maternal education empowers women to make informed decisions regarding their own health and their children’s well-being (40). It enhances health literacy, leading to better adherence to recommended practices. Additionally, educated mothers pass on knowledge and positive behaviors to their children, creating a cycle of improved health (38). Overall, promoting education among women is crucial for breaking the cycle of childhood underweight and improving overall health outcomes.

Significantly, the likelihood of underweight increased as the household wealth index declined from richer to the poorest, with the richest group serving as the reference. This observation aligns with previous research (13, 41, 42). This could be due to the fact that children from poorer households are more likely to experience underweight due to limited access to resources such as nutritious food and healthcare (41). Wealthier households, on the other hand, can invest more in their children’s well-being (41). This suggests addressing wealth-related disparities is crucial for preventing childhood underweight and improving overall child nutrition.

We found that children born to mothers who had no or 1–3 antenatal care (ANC) visits during pregnancy were more likely to be underweight compared to children born to mothers who had four or more ANC visits. This finding aligns with previous research studies (43–46). One possible reason for this association is that access to health care services, such as ANC, serves as a crucial source of information for women regarding nutrition and overall health (45). Additionally, mothers who received ANC follow-ups had opportunities for knowledge sharing related to optimal infant and young child feeding (47). Consequently, children whose mothers had ANC visits were less likely to experience underweight. Furthermore, ANC visits provided mothers with essential information about breastfeeding, which plays an indispensable role in minimizing underweight among children (46).

Consistent with prior research (48–50), this study revealed that children born in communities with high poverty levels had elevated odds of being underweight compared to their counterparts born in low-poverty communities. This implies that childhood underweight is a critical issue, particularly in communities grappling with high poverty levels. Several factors contribute to this concerning phenomenon. First, families living in poverty often struggle to provide adequate nutrition for their children due to limited financial resources (51). Poor living conditions, a lack of clean water, and inadequate sanitation facilities also impact child well-being (14, 52). Additionally, food insecurity, inadequate healthcare access, and indoor pollution further exacerbate underweight rates (52). Therefore, addressing poverty, education, healthcare, and tailored interventions in communities with high poverty are essential to combating childhood underweight in impoverished communities (14).

This study also revealed a significant association between the low income level of countries and higher odds of underweight, particularly when compared to lower-middle income countries. This finding aligns with results from another studies (14, 53). The economic challenges faced by low-income countries can directly impact child growth and development due to limited access to essential resources. These constraints include inadequate access to nutritious food, clean water, sanitation, and healthcare services (54). Investing in childhood nutrition and ensuring access to these fundamental needs can play a pivotal role in reducing underweight rates. By addressing these critical factors, we not only improve children’s health but also create a pathway for their future economic opportunities (54, 55).

The findings of this study should be regarded with the following limitations: First, it relies on data from standard demographic and health surveys conducted in specific low and lower-middle-income African countries, potentially introducing sampling bias. Second, the cross-sectional design captures data at a single point in time, limiting insights into causal relationships. Third, self-reported data may be subject to recall bias.

## Conclusion

The high prevalence of underweight among children under the age of five in low and lower-middle income African countries underscores the need for targeted interventions. By addressing individual, community, and country-level factors, we can work toward improving child nutrition and well-being.

## Data Availability

The datasets presented in this study can be found in online repositories. The names of the repository/repositories and accession number(s) can be found in the article/supplementary material.
